# Functions of double‐negative B cells in autoimmune diseases, infections, and cancers

**DOI:** 10.15252/emmm.202217341

**Published:** 2023-06-05

**Authors:** Michael King Yung Chung, Lanqi Gong, Dora Lai‐Wan Kwong, Victor Ho‐Fun Lee, Ann Wing‐Mui Lee, Xin‐Yuan Guan, Ngar‐Woon Kam, Wei Dai

**Affiliations:** ^1^ Department of Clinical Oncology, Li Ka Shing Faculty of Medicine The University of Hong Kong Hong Kong Hong Kong; ^2^ Department of Clinical Oncology, Shenzhen Key Laboratory for Cancer Metastasis and Personalized Therapy The University of Hong Kong‐Shenzhen Hospital Shenzhen China; ^3^ Laboratory for Synthetic Chemistry and Chemical Biology Hong Kong (SAR) China

**Keywords:** autoimmune disease, cancer immunosuppression, COVID‐19, double‐negative B cells, tumor microenvironment, Immunology

## Abstract

Most mature B cells can be divided into four subtypes based on the expression of the surface markers IgD and CD27: IgD^+^CD27^−^ naïve B cells, IgD^+^CD27^+^ unswitched memory B cells, IgD^−^CD27^+^ switched memory B cells, and IgD^−^CD27^−^ double‐negative (DN) B cells. Despite their small population size in normal peripheral blood, DN B cells play integral roles in various diseases. For example, they generate autoimmunity in autoimmune conditions, while these cells may generate both autoimmune and antipathogenic responses in COVID‐19, or act in a purely antipathogenic capacity in malaria. Recently, DN B cells have been identified in nasopharyngeal carcinoma and non‐small‐cell lung cancers, where they may play an immunosuppressive role. The distinct functions that DN B cells play in different diseases suggest that they are a heterogeneous B‐cell population. Therefore, further study of the mechanisms underlying the involvement of DN B cells in these diseases is essential for understanding their pathogenesis and the development of therapeutic strategies. Further research is thus warranted to characterize the DN B‐cell population in detail.

## Graphical abstract

This review discusses the functions of double‐negative B cells in different diseases.

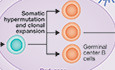

**Glossary**
Adaptive immunity: A type of immune response that can form long‐lasting immunological memory after encountering pathogens allowing them to quickly respond against those pathogens upon reinfection and generate sterilizing immunity to eliminate the pathogen before their replication. Immune B and T cells are involved in this process.
Affinity maturation: The general process by which B cells develop high‐affinity antibodies specific to antigens. Includes somatic hypermutation class switching and clonal selection and occurs within the germinal center of the B‐cell follicles.
Antibody class‐switching: Another process of B‐cell development altering the type of antibody produced by individual B cells allowing B cells to produce antibodies that can bind to their antigens strongly.
Antigen: The “target” of antibodies produced by antibody‐secreting cells. By binding to their antigens on pathogens the antibodies could either mark them for immune destruction or present them to activate other immune cells.
B‐cell follicles: An area within lymphoid organs where B cells can develop. Inside B‐cell follicles B cells can form germinal centers, where they can undergo processes to promote their development into switched‐memory B cells or long‐lived antibody‐secreting plasma cells with the help of different immune cells.
Bulk RNA sequencing: A gene sequencing method that measures the average expression levels of individual genes within a sample. The results obtained by this technique can generate an overall picture of the gene expression of a sample.
Clonal selection: A process in which B cells coding for antibodies with varying antigen affinities compete to interact with antigen‐presenting immune cells such as follicular T helper cells and follicular dendritic cells. B cells that could successfully bind to the presented antibody can receive activation signals to replicate, while the B cells that fail the process die via apoptosis.
Cytokines and chemokines: Small proteins secreted by cells that can control the activity of other cells usually immune cells. While cytokines usually affect the general properties of cells, such as their activation or suppression, chemokines are involved in the movement of these cells. Although immune cells are not the only cell type that can secrete them, many cytokines and chemokines are secreted by immune cells during an immune response.
Deconvolution analysis: A computational method that uses data from a particular single‐cell RNA sequencing (scRNA‐seq) dataset to estimate the proportion of different cell types in another bulk RNA sequencing (bulk RNA‐seq) dataset. If both RNA‐sequencing methods are conducted on patients with known clinical parameters deconvolution analysis can be useful in estimating the association between the cell populations from the scRNA‐seq dataset and the clinical parameters of the patients sampled for the bulk RNA‐seq dataset.
Extrafollicular B‐cell reactions: A type of B‐cell reaction that involves the development of B cells into short‐lived antibody‐secreting cells outside the B‐cell follicles and germinal centers. A potential development site for extrafollicular B‐cell reactions includes the extrafollicular loci within the lymph node.
Extragerminal center compartments: Areas outside the germinal center within lymphoid organs where memory B cells can develop into antibody‐secreting cells. One example of this includes the subcapsular proliferative foci an area just outside the B‐cell follicle that contains proliferating cells.
Immune exhaustion: A phenomenon where adaptive immune cells have impaired immune function after long‐term exposure to antigens.
Nasopharynx: An anatomical location in the human head behind the nasal cavity.
Neoantigens: New proteins are displayed on the surface of cancer cells due to their genetic mutations. They can be recognized by immune cells to attack cancer cells.
Single‐cell RNA sequencing: A gene sequencing method that sequences the RNA expression of every individual cell within a sample. This method accounts for the differences in gene expression from individual cells and is popular among scientists to analyze different cell types within samples.
Somatic tumor mutation burden: The number of genetic mutations accumulated in the cancer cells within a tumor as they become cancerous. Somatic mutations refer to the DNA mutations acquired by the cells after fertilization.
T‐independent type II antigen: A type of polysaccharide antigen that can stimulate B‐cell activation without the assistance of T cells. In contrast to T‐independent type I, antigen another polysaccharide antigen that simulates B cells via Toll‐like receptor (TLR) signaling, T‐independent type II signaling requires B‐cell receptor engagement.

## Introduction

B cells are a key component of the adaptive immune response. They can differentiate into plasma cells to secrete pathogen‐neutralizing antibodies and proinflammatory cytokines (Pioli, 2019) or develop into memory B cells to provide long‐lasting protection (Batista & Harwood, 2009). Alternatively, they can present antigens to activate other immune cells (Hong *et al*, 2018). The myriad of functions of B cells cements their status as an integral part of the human immune system. Human “mature B cells,” which have passed the B‐cell selection progress in the bone marrow and spleen (Rolink *et al*, 2001), can be categorized into different subsets based on their expression of the surface markers IgD and CD27 (Li *et al*, 2021). IgD is an antibody expressed on the surface of antigen‐naïve B cells (Li *et al*, 2021), while CD27 expression on B cells indicates the expression of immunoglobin genes that underwent somatic hypermutation (SHM) in the germinal center (GC) within the B‐cell follicles of lymphoid organs, a process B cells endure to produce high‐affinity antigen‐specific antibodies (Klein *et al*, 1998). The four major mature B‐cell subsets in human blood include IgD^+^CD27^−^ antigen‐naïve B cells, IgD^+^CD27^+^ unswitched memory B cells that produce IgM antibodies in a T‐cell‐independent manner (Capolunghi *et al*, 2013) and migrate to B‐cell follicles, where they differentiate into GC B cells to further their development (Seifert *et al*, 2015), and IgD^−^CD27^+^ switched memory cells that mature into IgD^−^CD27^+^ antibody‐secreting cells (ASCs) upon activation to produce high‐affinity antibodies. They express class‐switched antibodies such as IgG, which have higher affinity than unswitched IgM antibodies (Mäkelä *et al*, 1970). IgD^−^CD27^−^ B cells are termed double‐negative (DN) B cells and will be the focus of this review (Li *et al*, 2021).

Consistent with what their lack of CD27 expression may suggest, DN B cells underwent less SHM than their CD27‐expressing counterparts. Fecteau *et al* (2006) compared IgG‐producing DN B cells and switched memory B cells from healthy individuals and detected significantly lower mutation levels on the IgG transcripts from IgG^+^ DN B cells compared with IgG^+^ switched memory B cells. Nevertheless, DN B cells have undergone class‐switching and antigen selection (Fecteau *et al*, 2006) and have similar morphology and IGHV gene usage to switched memory cells (Wu *et al*, 2011). The mechanisms underlying their memory‐cell features despite their lack of CD27 expression remain elusive and have ignited speculation regarding DN B cells.

Double‐negative B cells are a rare B‐cell subset, making up approximately 5% of all peripheral B cells in healthy individuals (Li *et al*, 2021). However, DN B cells are involved in various diseases, including, but not limited, to systematic lupus erythematosus (SLE), COVID‐19, and malaria; low‐grade chronic inflammatory disorders, including aging; and certain cancers, such as non‐small‐cell lung cancer (NSCLC) and nasopharyngeal carcinoma (NPC) (Colonna‐Romano *et al*, 2009; Centuori *et al*, 2018; Jenks *et al*, 2018; Woodruff *et al*, 2020; Gong *et al*, 2021; Sutton *et al*, 2021). Detailed investigation of DN B cells in these diseases has suggested that DN B cells may represent a heterogeneous group of B cells with varying functions. While DN B cells are involved in the active immune response in SLE, chronic inflammatory diseases, and infectious diseases, current evidence regarding tumor‐infiltrating DN B cells implies that they may play an immunosuppressive role within the tumor microenvironment (TME) (Centuori *et al*, 2018; Gong *et al*, 2021). As DN B cells may be involved in various diseases, research on DN B cells will be essential in understanding these conditions and their treatments. For instance, therapies targeting DN B cells may improve clinical outcomes in severe COVID‐19, while understanding the reasons behind the enrichment of DN B cells in SLE may provide insights into SLE pathogenesis. Likewise, the characterization of potentially immunosuppressive tumor‐infiltrating DN B cells may contribute to the development of immunotherapeutic strategies in NSCLC and NPC. The function of DN B cells in different diseases is thus a topic warranting further exploration.

This review discusses the different subtypes of DN B cells in humans and their roles in different diseases, including autoimmune conditions, infectious diseases and cancers, and proposes hypotheses about the roles of DN B cells within the TME. The DN B cells discussed in this review are primarily defined using markers commonly found on human B cells.

## The different subtypes of “conventional” DN B cells

“Conventional” DN B cells, which refer to defined DN B‐cell populations reported in previous studies, can be divided into four subtypes: DN1 B cells, DN2 B cells, DN3 B cells, and DN4 B cells. Table 1 provides a summary of the phenotypes of the four subtypes of DN B cells, as well as their hypothetical functions.

**Table 1:** A summary of the four “conventional” DN B‐cell subsets.

“Conventional” DN B‐cell subsets				
Subtype	Defining markers	Additional markers	Possible function	Anatomical site
DN1	IgD^−^CD27^−^CXCR5^+^CD21^+^CD11c^−^IgE^−^	CD24^+^ T‐bet^−^ FCER2^−^ FCRL5^−^ CXCR3^−^	Switched‐memory B‐cell precursor	Blood
DN2	IgD^−^CD27^−^CXCR5^−^CD21^−^CD11c^+^IgE^−^	CD24^−^ T‐bet^+^ FCER2^−^ FCRL5^+^ CXCR3^+^	ASC precursor	
DN3	IgD^−^CD27^−^CXCR5^−^CD21^−^CD11c^−^IgE^−^	CD24^+^ T‐bet^+^ FCER2^−^ FCRL5^−^ CXCR3^−^	DN2 precursor	
DN4	IgD^−^CD27^−^CXCR5^+^CD21^+^CD11c^−^IgE^+^	CD24^+^ T‐bet^−^ FCER2^+^ FCRL5^−^ CXCR3^−^	IgE^+^ Switched‐memory B‐cell precursor	

The first subtype is DN1 B cells, which comprise the majority of the DN B population in healthy individuals (Jenks *et al*, 2018). DN1 B cells express the surface proteins CD21 and CXCR5 (Jenks *et al*, 2018). CD21 is a B‐cell coreceptor that works with the surface protein CD19 to enhance B‐cell receptor (BCR) signaling and antigen presentation to T cells (Cherukuri *et al*, 2001), while CXCR5 is the receptor for the chemokine CXCL13 and is important for B‐cell migration into germinal centers within the B‐cell follicles of lymphoid organs, where the follicular B‐cell response takes place (Kazanietz *et al*, 2019). During the follicular B‐cell response, B cells interact with other immune cells, such as follicular‐dendritic cells or follicular helper‐T cells, to undergo antigen affinity maturation, such as SHM, antibody‐class switching, and clonal selection (De Silva & Klein, 2015). B cells in the follicular reaction can develop into two cell types: long‐lived plasma cells that secrete high‐affinity IgG antibodies (De Silva & Klein, 2015) and switched‐memory B cells that can re‐enter the GC to further undergo SHM (McHeyzer‐williams *et al*, 2015) or directly differentiate into plasma cells upon antigen stimulation (Kometani *et al*, 2013; Akkaya *et al*, 2020). Current evidence suggests that DN1 B cells may act as precursors to switched memory B cells. While Jenks *et al* (2018) found similarities between the gene signatures of DN1 B cells and switched‐memory B cells, Stewart *et al*'s (2021) pseudotime analysis implicated DN1 B cells as a potential predecessor to IgD^−^CD27^+^ memory B cells. It could be reasonable to hypothesize that DN1 B cells may belong to the follicular pathway as well; however, further investigation is needed to confirm the above hypotheses.

DN2 B cells are the most well‐characterized DN B‐cell subtype. They do not express CD21 and CXCR5 (Jenks *et al*, 2018) and lack the expression of CD24, which is expressed on memory B cells and DN1 B cells but poorly in ASCs (Mensah *et al*, 2018; Stewart *et al*, 2021). DN2 B cells are characterized by their expression of CD11c (Jenks *et al*, 2018), a component of the complement receptor that can bind to the complement fragment iC3 to induce phagocytosis (Wu *et al*, 2018). Although the receptor is normally expressed by dendritic cells, some B cells express CD11c, and CD11c^+^ B cells have an increased capability for plasma cell differentiation compared with CD11c^−^ B cells (Golinski *et al*, 2020). DN2 B cells also express the transcription factor T‐bet, which promotes IgG2a class switching and survival of memory B cells (Peng *et al*, 2002). Another protein differentially expressed in DN2 cells is FCRL5 (Jenks *et al*, 2018). It encodes an IgG‐binding protein that alone inhibits BCR signaling (Haga *et al*, 2007) but can cooperate with CD21 to enhance BCR signaling (Franco *et al*, 2018). Moreover, DN2 B cells express CXCR3 on their surface (Woodruff *et al*, 2020). CXCR3 expression on B cells is induced by IFN‐y signaling and allows for travel via CXCL9‐mediated chemotaxis (Muehlinghaus *et al*, 2005). *In vitro* experiments have identified a population of IgD^+^CD27^−^CD21^−^CD11c^+^ “activated‐naïve” B cells, which are derived from naïve B cells exposed to proinflammatory factors such as TLR7 signaling, IFN‐y signaling, and IL‐21 signaling, as a developmental precursor for DN2 B cells (Jenks *et al*, 2018).

A common hypothesis regarding the development of DN2 B cells is that they may be a part of the extrafollicular B‐cell responses. This involves the GC‐independent development of B cells into short‐lived antibody‐secreting cells (ASCs) out of the B‐cell follicles (Elsner & Shlomchik, 2020) and can be divided into three possible types: T‐independent extrafollicular response elicited by T‐independent type II antigens (Jenks *et al*, 2019); T‐dependent extrafollicular response primed by BCL‐6^+^PD‐1^−^ T cells (Lee *et al*, 2011); and memory B‐cell reactivation in the extragerminal center compartments outside the GCs, such as the subcapsular proliferative foci (Moran *et al*, 2018; Beek *et al*, 2022). A summary of the types of extrafollicular reactions can be found in Table 2. Extrafollicular responses contribute to autoimmunity in mice with lupus‐like manifestations (William *et al*, 2002). Since DN2 B cells can differentiate into ASCs in SLE and display distinct gene expression from GC‐derived memory B cells (Jenks *et al*, 2018), they are hypothesized to be extrafollicular in nature. However, the development site and pathways for human DN2 B cells remain unknown, and studies focusing on DN2 B‐cell development are needed to assess whether DN2 B cells are truly extrafollicular B cells.

**Table 2:** Summary of different types of extrafollicular responses.

Types of extrafollicular responses					
Name	Initial B‐cell state	Inducers	Developmental site	Class‐switched Status	References
T‐cell independent	Naïve B cells	T‐independent type II antigens	Extrafollicular foci	May or may not be class‐switched	Lortan *et al* (1992) and Jenks *et al* (2019)
T‐cell dependent		BCL‐6^+^ PD‐1^−^ T cells + IL‐21	T‐B border + extrafollicular foci	Class‐switching may occur, IgM and IgG antibodies are involved	Toellner *et al* (1998), Cunningham *et al* (2007) and Lee *et al* (2011)
Memory B‐cell reactivation	GC‐derived memory B cells	Antigen‐reencounter + helper T cells	Extragerminal center compartments including subcapsular proliferative foci	Class‐switched	Moran *et al* (2018) and Beek *et al* (2022)

Recent studies have also identified new DN B‐cell subtypes. One of these DN subtypes is the “DN3” subset, which lacks expression of both CD21 and CD11c (Sosa‐Hernández *et al*, 2020; Woodruff *et al*, 2020). Although DN3 B cells are yet to be fully characterized, Stewart *et al*'s (2021) study on different DN B‐cell subsets suggested that they may be predecessors of DN2 B cells, as trajectory analysis identified a strong developmental flow from DN3 to DN2 B cells. The potential role of DN3 B cells as a possible precursor of DN2 B cells is supported by their similar gene expression. For example, both DN2 and DN3 B cells lack *CXCR5* expression and express T‐bet, although DN3 B cells express T‐bet at a lower level (Stewart *et al*, 2021). However, DN3 cells express CD24, while DN2 B cells do not (Stewart *et al*, 2021). Although both DN3 B cells and “activated naïve B cells” are potential predecessors of DN2 B cells, the genotype of both B‐cell populations is distinct. For example, “activated naïve” B cells express CD11c, which DN3 cells do not express (Jenks *et al*, 2018; Stewart *et al*, 2021). DN3 B cells and “activated naïve B cells” may therefore belong to different developmental stages or lineages in the DN2 B‐cell developmental pathway, with studies uncovering the developmental stages and relationships of both precursors being of value in fully understanding the development of DN2 B cells.

Stewart *et al* (2021) also identified an IgE‐enriched DN B population, which they considered a novel DN subpopulation termed DN4. As IgE antibodies are mainly involved in allergic reactions (Gould & Sutton, 2008), DN4 B cells were suspected to be involved in allergic responses, and their enrichment was hypothesized to be triggered by prior allergen exposure (Stewart *et al*, 2021). Pseudotime analysis suggested that DN4 B cells may be precursors to IgE‐expressing memory B cells, such as DN1 B cells, with both DN subpopulations expressing CXCR5, CD24, and CD21 (Stewart *et al*, 2021). Interestingly, DN4 B cells differentially express *FCER2/*CD23 (Stewart *et al*, 2021), an IgE receptor that downregulates BCR signaling (Liu *et al*, 2016), while their differential expression of the BCR coactivator CD40 indicates that they may be in an activated state in patients suffering from allergic responses (Stewart *et al*, 2021). However, caution is needed when interpreting these results, as Stewart *et al*'s (2021) study was limited by a small sample size, which only included one subject and two additional subjects for validation. The fact that DN4 B cells were discovered in one subject who suffered from an allergic response shortly before the study (Stewart *et al*, 2021) has only raised more issues. As the properties of B cells may be affected as a result of the allergic response, as seen by the dramatic increase in IgE production of B cells from allergic nasal mucosa compared with healthy nasal mucosa (KleinJan *et al*, 2000), the DN B population may have only gained their specific gene expression features due to the allergic response and otherwise may not express them. Additional caution is hence encouraged when looking at this potential DN subset, and more studies with larger sample sizes are needed to fully confirm the existence and characterize the features of DN4 B cells if they do exist.

**Figure 1:**
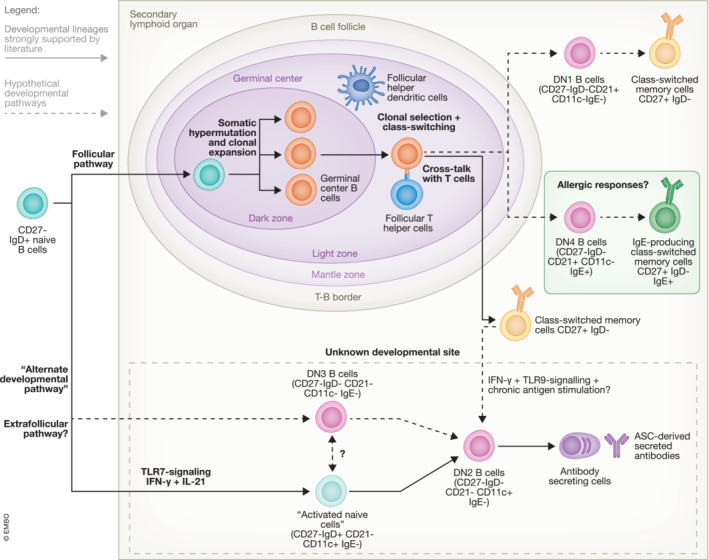
Hypothesized trajectories of B‐cell development into different subtypes of DN B cells DN1 and DN4 B cells are suspected to be precursors of class‐switched memory cells (Stewart *et al*, 2021). Moreover, DN2 B cells can differentiate into ASCs, and “activated naïve” B cells plus DN3 B cells have been suggested as potential DN2 B‐cell precursors (Jenks *et al*, 2018; Stewart *et al*, 2021). Although DN2 B cells have been suggested to be on an alternate developmental pathway from switched memory B cells (Jenks *et al*, 2018), GC‐derived memory B cells may gain features associated with DN2 B cells under inflammatory conditions and develop into DN2‐like “atypical B cells” in malaria, which will be discussed further below (Ambegaonkar *et al*, 2019; Holla *et al*, 2021). Due to the ambiguity surrounding the developmental site of DN2 B cells, the above findings surrounding “atypical B cells” opens the possibility of GC involvement in DN2 B development. More research is needed to fully uncover the developmental pathways of different DN B cells.

In summary, four DN B‐cell subsets have been identified in previous studies, and they can be classified using various markers, such as CD21, CXCR5, CD11c, T‐bet, and IgE. Figure 1 summarizes the hypothetical pathways that naïve B cells can utilize to develop into these four subtypes of DN B cells and possible terminal differentiation states that DN B cells can develop into. However, as DN B cells are relatively not well‐characterized in an overall sense, information regarding many DN B‐cell subpopulations remains scarce, which prevents them from being characterized using those markers. This includes DN B cells present in NPC and NSCLC, for which there are few data on their gene signatures and surface marker expression, let alone their precise roles within their respective malignancies (Centuori *et al*, 2018; Gong *et al*, 2021). Furthermore, the DN B population discovered in blood samples from elderly humans by Colonna‐Romano *et al* (2009) showed downregulated expression of the anti‐apoptotic gene BCL2 and antigen‐presenting genes such as HLA‐DR and CD40. However, the expression patterns of genes not mentioned above remain elusive, and the available data may not be sufficient for classifying them. Finally, using the markers above to classify DN B cells may not be sufficient to distinguish between DN2 B cells and the “atypical B‐cell” DN B subset in malaria (Sutton *et al*, 2021). Both DN B subpopulations express CD11c and T‐bet and lack the expression of CXCR5 and CD21 (Jenks *et al*, 2018; Sutton *et al*, 2021). As a result, “atypical B cells” may be considered analogous to DN2 B cells by examining their marker expression. However, some differences exist between these cells. For example, DN2 B cells have few somatic mutations compared with “atypical B cells,” which have somatic mutation levels comparable to those of switched‐memory B cells (Jenks *et al*, 2018; Sutton *et al*, 2021). The ambiguity surrounding whether “atypical B cells” and DN2 B cells should be considered as the same or distinct cell types cannot be addressed by the current classification system, and further studies on the different DN B‐cell subtypes are needed for a comprehensive DN B‐cell subset classification.

## An overview of DN B‐cell involvement within diseases

The current literature suggests that DN B cells participate in various processes, through which they take part in autoimmunity, act as a double‐edged sword generating both autoimmune and antipathogenic immune responses, or function purely to defend the host against pathogens, depending on the disease (Jenks *et al*, 2018; Woodruff *et al*, 2020; Sutton *et al*, 2021). DN B cells can also be found in tumors, and current evidence suggests that tumor‐infiltrating DN B cells may reflect the immunosuppressive nature of the TME (Centuori *et al*, 2018; Gong *et al*, 2021). This subsection aims to explore the nature of DN B‐cell involvement in the disease types mentioned above, and Table 3 below summarizes the different subtypes of DN B cells involved in various conditions and their possible roles.

**Table 3:** Different DN B subtypes involved in various diseases and their hypothetical roles.

Type of DN B cells involved in human diseases			
Diseases	DN subtypes involved	Hypothetical role	Anatomical site
SLE	DN2 + DN3	Autoimmunogenic	Blood
Obesity	DN2	Autoimmunogenic	Blood
Aging	Unspecified DN subtype	Exhausted B cells	Blood
COVID‐19	DN2 + DN3	Autoimmunogenic + Anti‐pathogenic	Blood
Malaria	“Atypical B cells”	Anti‐pathogenic	Blood
NSCLC	Unspecified DN subtypes	Potentially immunosuppressive	Tumor tissue
NPC			

## DN B cells within autoimmune conditions

### Systemic lupus erythematosus (SLE)

Double‐negative B cells are implicated in various autoimmune conditions, with their involvement within SLE being the most well‐characterized. In SLE patients, autoantibodies targeting nucleic acids are produced, while the removal of nucleic acid‐containing apoptotic cell debris is impaired, resulting in the persistence of immune complexes capable of triggering autoimmune reactions (Kaul *et al*, 2016). Different B‐cell populations, including ASCs and DN B cells, are involved in the pathogenesis of the disease through the secretion of autoantibodies. DN B cells are enriched within SLE patients, and they correlate with a high SLE disease activity score (Wei *et al*, 2007). DN B cells may be involved in lupus nephritis, the renal manifestation of SLE, where the kidney is damaged due to the deposition of autoantibodies in glomerular sites (Anders *et al*, 2020). The frequency of DN B cells was positively correlated with the incidence of lupus nephritis and increased 24‐h urine protein excretion levels in SLE patients (You *et al*, 2020). Since 24‐h urine protein extraction levels are commonly used to estimate the degree of proteinuria and therefore renal damage within SLE patients (Anders *et al*, 2020), this association suggests that DN B cells may play a role in renal damage in SLE.

The autoimmune involvement of DN B cells in SLE is further supported by evidence of their “activated” state in the disease. A feature displayed by DN B cells in SLE is their cytoplasmic localization of FOXO1. FOXO1 is a transcription factor that normally resides within the nucleus and plays a key role in B‐cell development, with FOXO1 deficiency within peripheral B cells reducing their migration to secondary lymphoid organs and impairing their ability to undergo class‐switching (Dengler *et al*, 2008). Hritizo Ahye and Golding discovered that FOXO1 was mostly located within the cytoplasm instead of the nucleus in SLE‐patient‐derived DN B cells (Hritzo Ahye & Golding, 2018). Those DN B cells were in an activated state, as evidenced by their reduced expression of CD20 and increased CD95 expression (Hritzo Ahye & Golding, 2018). CD95 expression is induced after B‐cell activation (Catlett & Bishop, 1999) and upregulated in the DN B‐cell population in SLE (Jacobi *et al*, 2008). However, as CD95 can induce apoptosis by binding to the FADD protein (Catlett & Bishop, 1999), targeting DN B cells in SLE by inducing CD95‐mediated apoptosis may be a valid therapeutic strategy.

Jenks *et al* (2018) provided a more comprehensive study of the DN B‐cell population in SLE. The vast majority of the DN population enriched in SLE was identified as CXCR5^−^CD21^−^ CD11c^+^ DN2 B cells, which occupied the majority of nonplasmablast CD19^+^ B cells within the disease. The presence of IFN‐y, IL‐21, and TLR7 signaling was sufficient to give rise to DN2 B cells and their precursor, “activated naïve” cells, from naïve B cells and was essential for the development of DN2 B cells into autoreactive ASCs (Jenks *et al*, 2018). DN2 B cells were also found to be hyperresponsive to TLR7 signaling (Jenks *et al*, 2018). A potential explanation for the enrichment of DN 2 B cells in SLE is that B cells may have enhanced access to the inflammatory signals required for DN2 B‐cell generation. Genetic alterations in TLR7 function have been identified as risk factors for SLE. Brown *et al* (2022) identified a gain‐of‐function mutation of TLR7 in SLE patients that induces autoimmunity and the accumulation of DN2‐like “age‐associated B cells” within mice. The deletion of XIST, a key player in X chromosome inactivation capable of silencing genes on the X chromosome, such as *TLR7* (Souyris *et al*, 2018), in human B cells can drive the accumulation of DN2 B cells *in vitro* (Yu *et al*, 2021) by causing *TLR7* from both X chromosomes to be expressed in DN2 B cells. Moreover, blood IFN‐y levels are positively correlated with high SLE disease activity and active nephritis (Oke *et al*, 2019), which are associated with DN B cells in SLE (Wei *et al*, 2007; You *et al*, 2020). Systematic lupus erythematosus patients were also observed to have an increased population of IL‐21‐expressing T cells (Dolff *et al*, 2011). Therefore, the chronic inflammatory landscape of SLE may provide an optimal environment for B cells to develop into DN2 B cells.

Finally, DN3 B cells are also enriched within SLE (Jenks *et al*, 2021), consistent with their proposed role as DN2 B‐cell precursors; however, other than a study reporting their increased frequency in SLE patients treated with the B‐cell depleting antibody rituximab (Faustini *et al*, 2022), the involvement of DN3 cells within the pathogenesis and treatment of SLE is yet to be uncovered.

To conclude, DN2 B cells may play a significant role in SLE autoimmunity by developing into autoreactive ASCs. Moreover, studies investigating their use as therapeutic targets, such as by administrating TLR7 inhibitors or by inducing CD95‐mediated apoptosis, may reveal valid therapeutic strategies for SLE. Furthermore, future research on SLE can focus on the role of DN3 B cells within SLE and assess their utility as therapeutic targets to boost our understanding of autoimmune disease.

### Obesity

In obesity, macrophages are recruited into adipose tissues and stimulated through various mechanisms, including damage‐associated molecular patterns released from dying adipocytes and free fatty acids in the blood to activate TLR4 signaling (Reilly & Saltiel, 2017). This causes the secretion of chemokines and proinflammatory cytokines into the bloodstream (Russo & Lumeng, 2018), recruiting and activating other immune cells in adipose tissue. Some of these proinflammatory cytokines in obesity include IFN‐y, secreted by CD4^+^ T cells upon receiving antigen presentation from adipocytes (Bradley *et al*, 2022), and IL‐21, which is upregulated in adipose tissues from obese mice (Fabrizi *et al*, 2014). Mice fed a high‐fat diet have increased production of nucleic acid‐containing extracellular traps by neutrophils and macrophages, which can activate adipose macrophages via TLR7 signaling (Revelo Xavier *et al*, 2016). As B cells are involved in the inflammatory landscape present in obesity, undergoing expansion in adipose tissues from mice fed a high‐fat diet, where they can activate T cells and macrophages (Winer *et al*, 2014; Zatterale *et al*, 2019), they have the potential to receive IL‐21, IFN‐y, and TLR7 stimulation, priming them to a DN2 B‐cell fate. Furthermore, CD11c^+^ T‐bet^+^ DN2 B cells are elevated in the blood of obese individuals compared to lean individuals (Frasca *et al*, 2021) and may be involved in autoimmune responses in obesity. DN2 population frequency in obese subjects positively correlates with autoantibody production, and their depletion is associated with a decrease in antibody titer (Frasca *et al*, 2021). Since antibodies can form immune complexes to activate macrophages in mice while autoantibody production is linked to insulin resistance, a symptom of obesity (Winer *et al*, 2011), DN2 B cells may contribute to the pathogenesis of obesity, and research should be conducted to elucidate their role within the chronic inflammatory landscape of this condition.

### Aging

Aging weakens the immune system. The overall B‐cell population in elderly humans has been observed to decline over age, with B cells derived from elderly blood having reduced AID expression (Frasca *et al*, 2008). Since AID is an enzyme important for immunoglobulin class‐switching for B cells that results in the production of stronger‐affinity antibodies (Muramatsu *et al*, 2000), these B cells have defects in antibody class‐switching (Frasca *et al*, 2008). Consistent with the functional defects reported in aged B cells, Colonna‐Romano *et al* (2009) identified an unspecified subset of DN B cells in elderly humans, which they speculated to be exhausted B cells due to their reduced function. The DN B cells they discovered had short telomere lengths and low BCL2 expression, suggesting susceptibility to apoptosis (Colonna‐Romano *et al*, 2009), while their low expression of CD80, HLA‐DR, and CD40 suggested a reduced antigen‐presenting capability, implying a reduced ability to activate other immune cells (Colonna‐Romano *et al*, 2009). However, many features regarding the DN B‐cell population have yet to be elucidated, such as their surface marker expression pattern and the genes that may be enriched in this population. Therefore, more research is needed to further characterize the DN B population and uncover the circumstances giving rise to the cell type in elderly individuals.

Despite the age‐associated decline in immune function, aging can be considered a chronic inflammatory condition to some extent, as aging bodies are constantly in a state of chronic low‐grade inflammation characterized as “inflammaging.” This process is mediated by the innate immune response, activated by stimuli such as cell debris and interactions with the aging gut microbiota (Franceschi *et al*, 2018). However, the innate immune system is not the only player in “inflammaging,” as adaptive immune cells such as T cells may be involved. CD4^+^ T cells from aged humans have enhanced proliferation and activation due to cytoplasmic DNA accumulation and are differentially enriched in autoimmunity‐related genes compared with those from young subjects (Wang *et al*, 2021). DN B cells may also be involved in “inflammaging.” Rubtsov *et al* (2011) discovered a murine B‐cell population termed “age‐associated B cells” (ABCs) that was enriched in aged mice. “Age‐associated B cells” are similar to human DN2 B cells, as they both express CD11c and T‐bet and can be activated through TLR7 signaling (Rubtsov *et al*, 2011, 2015). Age‐associated B cells were hypothesized to play an autoimmune role within aged mice, as they were noted to be potent antigen‐presenting cells and were capable of producing autoantibodies *in vivo* (Rubtsov *et al*, 2011, 2015). As aged individuals possess higher frequencies of autoantibodies than younger people (Manoussakis *et al*, 1987), while DN2 B cells are associated with autoantibody production (Jenks *et al*, 2018; Frasca *et al*, 2021), research focusing on how DN2 B cells play an autoimmune role in elderly humans similar to that of DN2‐like ABCs in mice would be of interest.

## DN B cells within infectious diseases

Not only do DN B cells play a role in conditions involving autoimmunity and chronic inflammation, but recent studies have also found DN B‐cell populations to be enriched in infectious diseases, such as COVID‐19 and malaria (Woodruff *et al*, 2020; Sutton *et al*, 2021). While DN B‐cell populations may play both autoimmune and antipathogenic roles in COVID‐19, DN B cells in malaria function to defend the host against the pathogen. This subsection aims to explore the origins and roles of DN B cells in both diseases.

### COVID‐19

COVID‐19 is an acute respiratory disease caused by the beta‐coronavirus SARS‐CoV‐2 and since 2020 has been responsible for a global pandemic (Hu *et al*, 2021). Severe cases of COVID‐19 usually present with “cytokine storms,” an aggressive inflammatory response characterized by increased production of proinflammatory cytokines causing severe lung damage (Huang *et al*, 2020). Cytokine storms are triggered by SARS‐CoV‐2 infection of lung epithelial cells, causing them to secrete cytokines and chemokines to recruit immune cells to the lung tissue. Once arriving at the site of infection, immune cells are further stimulated to secrete proinflammatory cytokines, ultimately resulting in further lung damage (Yang *et al*, 2021). IFN‐y is among the various cytokines produced en masse, with plasma levels of the cytokine increased in the blood of COVID‐19 patients who require intensive care (Huang *et al*, 2020), while B cells can recognize single‐stranded RNA viruses, including coronaviruses such as SARS‐CoV‐2, via TLR7 signaling (Lund *et al*, 2004; V'Kovski *et al*, 2021). Since TLR7 and IFN‐y signaling are two of the three signaling pathways required for DN2 B‐cell development (Jenks *et al*, 2018), although the involvement of IL‐21 in the COVID‐19 cytokine storm has yet to be studied, viral infection may provide an environment conducive for the generation of DN2 B cells.

Woodruff *et al* (2020) discovered that severe COVID‐19 patients had enriched DN2 B‐cell populations compared with mild COVID‐19 patients and healthy controls, consistent with the expansion of ASCs such as plasmablasts in severe COVID‐19 (Kuri‐Cervantes *et al*, 2020; Woodruff *et al*, 2020). DN3 B cells were first discovered in COVID‐19 patients and were also found to be enriched in severe and critical COVID‐19 patients (Sosa‐Hernández *et al*, 2020; Woodruff *et al*, 2020). Similar to DN2 B cells, DN3 B cells may play a role in the pathogenesis of COVID‐19, as their frequency was negatively correlated with several ventilatory parameters, such as respiratory rate and oxygen saturation levels, in patients (Sosa‐Hernández *et al*, 2020) and positively correlated with proinflammatory features, such as high blood neutrophil/leukocyte count and high blood chemokine concentration (Cervantes‐Díaz *et al*, 2022). The evidence above implicates DN2 and DN3 B cells with severe COVID‐19 and poor prognosis. Owing to the similarity behind the B‐cell responses in COVID‐19 and SLE (Woodruff *et al*, 2020), the involvement of DN2 and DN3 B cells in autoimmune responses may provide a plausible explanation behind the association of DN B cells with poor prognosis in COVID‐19. Emerging evidence has supported the notion that B cells may be involved in COVID‐19‐associated autoimmunity, as high ASC content in COVID‐19 patients was associated with an enrichment of autoantibodies (Schultheiss *et al*, 2021). Woodruff *et al* (2022) also characterized a population of IgG1‐producing ASCs in severe COVID‐19 patients and discovered that some of the antibodies produced by those ASCs bound to both SARS‐CoV‐2 antigens and self‐antigens, suggesting that the ASCs above can be both antipathogenic and autoimmune in nature. Those ASCs enriched in severe COVID‐19 may originate from DN2 B cells owing to the low mutation frequencies on the ASC‐derived IgG1 antibodies (Woodruff *et al*, 2022), which is consistent with the reduced IgG mutation rate seen in DN2 B cells and DN2‐derived ASCs from SLE patients (Jenks *et al*, 2018). Finally, the DN3 B‐cell population was positively correlated with the autoreactive antibody titer of COVID‐19 patients (Castleman *et al*, 2022), further supporting the involvement of DN B cells in the autoimmunity surrounding severe COVID‐19. As autoantibodies were discovered in patients with post‐COVID‐19 syndrome, which involves the persistence of COVID‐19 symptoms after recovery from COVID‐19 (Rojas *et al*, 2022); research focusing on identifying the possible roles of DN B cells in post‐COVID‐19 syndrome would be of interest.

### Malaria

Malaria is caused by *Plasmodium* parasites, which are carried by anopheline mosquitos and can infect hepatocytes to generate merozoites (Phillips *et al*, 2017; Sato, 2021). The merozoites will then proceed to infect red blood cells, which produce malaria endotoxins to stimulate immune cells via Toll‐like receptor‐9 (TLR‐9) signaling (Phillips *et al*, 2017). As TLR‐9 signaling can cause unswitched‐memory B cells to differentiate into CD27^−^IgD^−^ B cells (Torigoe *et al*, 2017), DN B cells may be potentially involved in the antimalarial immune response.

A CD27^−^CD21^−^ B‐cell population termed “atypical B cells” was found in blood samples from malaria patients and those with prior exposure to *Plasmodium* antigens (Portugal *et al*, 2015) and was confirmed to be double‐negative for IgD and CD27 by recent studies (Sutton *et al*, 2021; Hopp *et al*, 2022). Current evidence has suggested that the “atypical B‐cell” population may have undergone affinity maturation in the germinal center, as evidenced by their comparative SHM level with IgD^−^ CD27^+^ switched memory B cells (Sutton *et al*, 2021). This finding contrasts with the low level of SHM observed in DN B cells in general, including DN2 B cells (Fecteau *et al*, 2006; Jenks *et al*, 2018). Nevertheless, the “atypical B‐cell” population expresses genes associated with DN2 B cells, such as *ITGAX*/CD11c, *TBX21*/T‐bet, and *FCRL5* (Kim *et al*, 2019; Sutton *et al*, 2021; Hopp *et al*, 2022). Single‐cell pseudotime analysis suggests that the DN B‐cell subpopulation may belong to an alternative developmental pathway and in a further developmental state compared with “classical” switched‐memory B cells (Sutton *et al*, 2021), opening up the possibility that the gain of DN2‐B‐associated genes may have occurred in a later developmental event than affinity maturation. The “atypical B‐cell” DN subtype was initially reported to have impaired BCR signaling and reduced *in vitro* function (Portugal *et al*, 2015). However, emerging evidence suggests that they could be involved in the immune response against malaria. Furthermore, recent studies have provided evidence that the DN B‐cell population can respond to BCR signaling from membrane‐associated antigens but not soluble antigens (Ambegaonkar *et al*, 2020) and that their seemingly impaired BCR signaling may be due to an increased antigen affinity threshold for activation, as IgD^−^CD27^−^ “atypical B cells” respond less well to low‐affinity antigens (Holla *et al*, 2021). During acute malaria, the “atypical B cells” upregulate TLR9 expression (Hopp *et al*, 2022), while their increased expression of the antigen‐presenting HLA‐class I and class II proteins allows them to present antigen well (Hopp *et al*, 2022). Finally, the “atypical B cells” can produce immune memory, with “atypical B cells” from mice previously infected with *Plasmodium* 3 months ago responding to restimulation to produce antibodies *ex vivo* (Kim *et al*, 2019 *)*.

Studies investigating the origin of “atypical B cells” have proposed hypotheses about their developmental origin. Naïve and memory B cells cultured with IFN‐y and TLR9 agonists under chronic antigen engagement expressed “atypical B‐cell”‐like features such as T‐bet, FCRL5, CD11c, and CXCR3, implicating IFN‐y, TLR9 signaling and chronic antigen stimulation as potential stimulations inducing “atypical B‐cell development” (Ambegaonkar *et al*, 2019). IFN‐y production was increased in mice infected with *Plasmodium* parasites to promote TLR‐mediated immune responses against malaria (Franklin *et al*, 2009), while prolonged antigen exposure could have occurred due to the difficulty of obtaining sterilizing immunity against Plasmodium infection despite repeated infections, as exemplified by the lack of change in the *P. falciparum* infection rate with age (Tran *et al*, 2013). Although both naïve B cells and memory B cells gain “atypical B‐cell” features, only memory B cells induced under the above conditions have downregulated CD21 expression in the DN B‐cell subset (Ambegaonkar *et al*, 2019), suggesting that memory B cells may be a more immediate precursor to “atypical B cells.” Holla *et al* (2021) conducted pseudotime analysis on different B cells from adults exposed to malaria, and their findings suggested that IgD^−^CD27^+^ memory B cells may be a close developmental precursor to “atypical B cells”.

Taken together, the above data suggest that “atypical B cells” may develop from switched memory B cells under proinflammatory conditions. However, more research is needed to evaluate the hypotheses regarding “atypical B cells” and identify the circumstances regulating them in malaria, such as determining the reasons behind their downregulated CD27 expression and increased antigen affinity threshold for activation and their role in the difficulties of forming sterilizing immunity against malaria. Likewise, as “atypical B cells” share many similarities with DN2 B cells despite their differences, studies determining whether they belong to the same cell type and reconciling their difference are needed.

## The role of DN B cells in cancers

In the field of oncology, IgD^−^ CD27^−^ B cells have been identified in two types of cancers: non‐small‐cell lung cancer (NSCLC) and nasopharyngeal carcinoma (NPC) (Centuori *et al*, 2018; Gong *et al*, 2021). This subsection aims to introduce the two types of cancers in which DN B cells are involved and discuss the role of DN B cells within their TME.

Non‐small‐cell lung cancer is an umbrella term for a variety of subgroups of lung cancers, with the majority of cases belonging to the adenocarcinoma subtype, which has glandular histology and is located in the distal lung, while tumors of the squamous cell carcinoma subtype are located in the proximal airways (Chen *et al*, 2014). Due to the close association between smoking and NSCLC, cancer cells in NSCLC have a high somatic tumor mutation burden because of prolonged carcinogen exposure from cigarette smoke (Alexandrov *et al*, 2013). This means that NSCLC cancer cells might express relatively high amounts of neoantigens and may therefore be vulnerable to immune destruction, making the modulation of immune cells in the TME a viable strategy to treat NSCLC.

Double‐negative B cells are enriched in the NSCLC TME compared with normal lung tissues, making up approximately 0.2–8.5% of all live singlet cells derived from NSCLC patients (Centuori *et al*, 2018). Despite their low abundance and the lack of association between NSCLC‐infiltrating DN B cells and major clinical parameters, other than a positive correlation with the presence of moderately differentiated tumors (Centuori *et al*, 2018), DN B cells may be associated with immunosuppression within the NSCLC TME, as the DN B‐cell frequency was inversely correlated with that of IgD^−^CD27^+^ “affinity‐matured” B cells in the tumor (Centuori *et al*, 2018), which included switched memory B cells and ASCs such as plasmablasts and plasma cells (Sanz *et al*, 2019). This finding suggests that NSCLC‐infiltrating DN B cells may have a developmental relationship with IgD^−^CD27^+^ B cells in the NSCLC TME and potentially be associated with a decline in IgD^−^CD27^+^ B cells within NSCLC tumors. As early NSCLC patient‐derived plasma cells have tumor‐suppressing capabilities, while late NSCLC patient‐derived plasma cells can promote tumor growth in NSCLC cell lines (Chen *et al*, 2020), DN B cells may have varying functions in different NSCLC stages if they are developmentally related to plasma cells in the “affinity‐matured” B‐cell population. Research focused on further investigating the relationship between DN B cells and the cell subpopulations within the “affinity‐matured” B cells will be important.

Moreover, NPC is a subtype of head‐and‐neck cancer occurring in the nasopharynx. This type of cancer is endemic in South China, Southeast Asia, and North Africa (Wong *et al*, 2021), and it can be divided into keratinizing, nonkeratinizing, and basaloid squamous cell carcinoma subtypes, with the nonkeratinizing subtype further divided into “well‐differentiated” or “poorly differentiated” subtypes based on the tumor differentiation status (Wong *et al*, 2021; Badoual, 2022). Epstein–Barr virus (EBV) infection is highly associated with NPC, with the virus being detected in all NPC cases belonging to the nonkeratinizing subtype, the most common NPC subtype in endemic regions (Tsao *et al*, 2017). Epstein–Barr virus infection can activate the NF‐κB signaling pathway in epithelial cells within the nasopharynx causing them to secrete proinflammatory factors, such as the cytokines IL‐6 and IL‐8 (Eliopoulos *et al*, 1997; Koon *et al*, 2010), therefore creating a chronically inflamed environment shaping the NPC TME.

Under EBV‐induced chronic inflammation, different types of immune cells, such as plasma B cells and DN B cells, are recruited into the NPC TME from the surrounding blood circulation. NPC‐infiltrating DN B cells were first identified in Gong *et al*'s (2021) study, where the authors conducted scRNA‐seq on tissue biopsies obtained from NPC patients. Similar to the findings in the NSCLC study (Centuori *et al*, 2018), NPC tumors were enriched in DN B cells, occupying a median proportion of 5.3% out of the total B‐cell population from each patient, while they only appeared in negligible quantities in nasopharyngeal lymphatic hyperplasia tissue samples (Gong *et al*, 2021). The enrichment of DN B cells within the NPC TME was confirmed by a deconvolution analysis utilizing public bulk RNA sequencing data from NPC patients (Gong *et al*, 2021). Additionally, NPC‐infiltrating DN B cells interacted with exhausted T cells within the NPC TME via the CXCL13‐CXCR5 axis, suggesting that at least a portion of DN B cells expressed CXCR5 and were recruited into NPC tumors via CXCL13‐mediated chemotaxis. However, further investigation is needed to determine whether the CXCR5‐expressing DN B population corresponds to the DN1 subtype and elucidate the proportion of CXCR5^+^ DN B cells within the overall DN B‐cell population. Although Gong *et al*'s (2021) study was not solely focused on NPC‐infiltrating DN B cells and therefore did not fully characterize them, they discovered that the population size of those DN B cells within NPC patients was correlated with a worse clinical outcome. This finding implicates DN B cells as a potential immunotherapeutic target in NPC and calls for further investigation to uncover the functional role of NPC‐infiltrating DN B cells and the detailed mechanisms underlying their association with poor clinical outcomes in NPC.

## Research perspectives

Despite the emergence of studies focusing on DN B cells, this B‐cell subpopulation is still understudied and needs further investigation. For instance, creating a unifying term defining IgD^−^ CD27^−^ B cells may aid research toward DN B‐cell populations. In the literature, human DN B cells have been referred to by many names, including “double‐negative B cells,” “age‐associated B cells,” and “atypical B cells” (Jenks *et al*, 2018; Sutton *et al*, 2021; Brown *et al*, 2022). The lack of unifying nomenclature for DN B cells may cause confusion, which can be solved by creating an umbrella term for DN B cells.

Furthermore, the site of DN2 B cell development in humans is unknown. DN2 B cells have been suggested to be extrafollicular B cells that develop independently from GCs, as their distinct features compared with GC‐derived memory B cells suggest that DN2 B cells may lie on an alternative developmental branch (Jenks *et al*, 2018; Woodruff *et al*, 2020; Stewart *et al*, 2021). However, the exact developmental site and pathways of human DN2 B cells have yet to be elucidated. To add to the ambiguity of the mechanisms giving rise to DN2 B cells, GC‐derived memory B cells may gain DN2‐like features such as CD11c and T‐bet expression under proinflammatory signals and chronic antigen exposure, which might be the case for DN2‐like “atypical B cells” (Ambegaonkar *et al*, 2019; Holla *et al*, 2021; Sutton *et al*, 2021). The findings on “atypical B cells” open the possibility that the GC may be involved in DN2 B‐cell development contrary to what was implied previously, especially since memory B cells could develop into short‐lived ASCs upon restimulation (Moran *et al*, 2018). Although Song *et al* (2022) provided evidence of the germinal center‐independent development of a DN2‐like T‐bet^+^CD11c^+^ B‐cell population in mice, future studies are needed to evaluate whether this is the case for DN2 B cells in humans. Moreover, the highly overlapping gene signature between “atypical B cells” and DN2 B cells implies that they might belong to the same subpopulation despite their distinct terminology (Holla *et al*, 2021). Due to existing data supporting a GC‐derived memory B‐cell origin for “atypical B cells,” studies comparing both DN B subtypes to see whether they correspond to each other would provide valuable hints for understanding DN2 B‐cell development.

In contrast to the relatively high level of attention that DN2 B cells have received in past research studies, other DN B‐cell subsets have relatively not been well‐studied. For example, there are many questions regarding the precise developmental position of DN1 and DN3 B cells, with more functional studies being needed to fully confirm many of the hypotheses about them. This is also the case for DN B cells enriched in NSCLC and NPC samples and the unspecified DN population associated with aging. Current evidence implies that their function may be contrary to that of DN B cells in other diseases. While the former two may border immunosuppressive effects via cytokine release or cell–cell interactions or at least reflect the immunosuppressive nature of the TME (Centuori *et al*, 2018; Gong *et al*, 2021), the latter may reflect an age‐associated weakening of the immune system (Colonna‐Romano *et al*, 2009). Therefore, they may be different DN B‐cell populations compared to those cells previously characterized in the scientific literature, and future research focusing on the further characterization of those DN B cells and the conditions giving rise to them is needed. In addition, the implication of tumor‐infiltrating DN B cells with cancer immunosuppression means that they might be immunotherapeutic targets, which encourages the identification and characterization of DN B cells in the TMEs of other cancers.

The findings regarding human DN B cells suggest that they may represent a heterogeneous B‐cell population capable of playing varying functions in different conditions. Future studies are needed to characterize currently known DN B‐cell subtypes and identify DN B‐cell subtypes and their functions in other diseases.

## Author contributions

**Michael King Yung Chung:** Data curation; formal analysis; investigation; visualization; methodology; writing – original draft; writing – review and editing. **Lanqi Gong:** Writing – original draft; writing – review and editing. **Dora Lai‐Wan Kwong:** Writing – review and editing. **Victor Ho‐Fun Lee:** Writing – review and editing. **Ann Wing‐Mui Lee:** Writing – review and editing. **Xin‐Yuan Guan:** Funding acquisition; writing – review and editing. **Ngar‐Woon Kam:** Conceptualization; supervision; writing – original draft; writing – review and editing. **Wei Dai:** Conceptualization; resources; supervision; funding acquisition; methodology; writing – original draft; project administration; writing – review and editing.

## Disclosure and competing interests statement

The authors declare that they have no conflict of interest.

**Pending issues**

- Generate a unifying nomenclature for IgD^−^CD27^−^ DN B‐cell populations.
- Conduct more studies to characterize DN B cells to refine the current DN B classification system.
- Conduct functional studies to validate the hypothetical developmental positions of DN1‐DN4 subsets.
- Evaluate the site of development of DN2 and DN3 B cells in lymphoid tissues to determine if they belong to the follicular or extrafollicular B‐cell reaction.
- Conduct studies with larger sample sizes to confirm the presence of DN4 B cells identified by Stewart *et al* (2021).
- Determine if “atypical B cells” in malaria correspond to DN2 B cells and reconcile the differences between the two cell types.
- Further characterize the origin and features of the DN B‐cell population discovered by Colonna‐Romano *et al* (2009), which remains understudied at the time of writing.
- Conduct more in‐depth functional and bioinformatics studies uncovering the roles of tumor‐infiltrating DN B cells in NSCLC and NPC.
- Develop therapeutic regimens targeting pathways enriched in DN B cells in SLE, such as TLR‐7 and CD95 signaling.
- Investigate the roles of DN2 B cells in the chronic inflammatory landscape in obesity and determine if older individuals have enriched DN2 B cells consistent to findings on aged mice.
- Confirm the autoimmune capabilities of DN2 and DN3 B cells within severe COVID‐19 and conduct research to elucidate their involvement in post‐COVID syndrome.
- Conduct more in‐depth functional and bioinformatics studies uncovering the roles of tumor‐infiltrating DN B cells in NSCLC and NPC.
- Investigate the developmental trajectory of the “atypical B‐cell” DN subtype in malaria to understand the mechanisms behind their downregulation of CD27 and their increased antigen affinity threshold for activation.
- Identify and characterize novel subtypes of DN B cells and their roles in other diseases.

## For more information

- Introduction to single‐cell RNA‐sequencing (scRNA‐seq), a method used by many of the studies cited in the review. https://singlecell.broadinstitute.org/single_cell
- Author's website. The first author and corresponding author's team focus on understanding the tumor microenvironment of NPC. http://weidai‐lab.hku.hk/
- CDC fact sheet for SLE: https://www.cdc.gov/lupus/facts/detailed.html
- CDC fact sheet for Malaria: https://www.cdc.gov/parasites/malaria/
- CDC fact sheet for COVID‐19. https://www.cdc.gov/coronavirus/2019‐ncov/
- CDC fact sheet for “long COVID‐19,” a condition that may be linked to autoimmunity as suggested by this literature review: https://www.cdc.gov/coronavirus/2019‐ncov/long‐term‐effects/index.html
- A link to a CDC webinar relating to the aging immune system, which is discussed by this literature review: https://www2.cdc.gov/vaccines/ed/nvpo/archives/downloads/NVPO_9_26_2017.pdf
- National Cancer Institute fact sheet for Non‐Small Cell Lung Cancer (NSCLC): https://www.cancer.gov/types/lung/patient/non‐small‐cell‐lung‐treatment‐pdq
- National Cancer Institute fact sheet for Nasopharyngeal carcinoma (NPC): https://www.cancer.gov/types/head‐and‐neck/patient/adult/nasopharyngeal‐treatment‐pdq
- World Health Organization's report about obesity in Europe. Mentions health complications of obesity, such as inflammation: https://apps.who.int/iris/bitstream/handle/10665/353747/9789289057738‐eng.pdf

## References

[emmm202217341-bib-0001] Akkaya M , Kwak K , Pierce SK (2020) B cell memory: building two walls of protection against pathogens. Nat Rev Immunol 20: 229–238 31836872 10.1038/s41577-019-0244-2 PMC7223087

[emmm202217341-bib-0002] Alexandrov LB , Nik‐Zainal S , Wedge DC , Aparicio SA , Behjati S , Biankin AV , Bignell GR , Bolli N , Borg A , Børresen‐Dale AL *et al* (2013) Signatures of mutational processes in human cancer. Nature 500: 415–421 23945592 10.1038/nature12477 PMC3776390

[emmm202217341-bib-0003] Ambegaonkar AA , Nagata S , Pierce SK , Sohn H (2019) The differentiation *in vitro* of human tonsil B cells with the phenotypic and functional characteristics of T‐bet^+^ atypical memory B cells in malaria. Front Immunol 10: 852 31068937 10.3389/fimmu.2019.00852 PMC6491666

[emmm202217341-bib-0004] Ambegaonkar AA , Kwak K , Sohn H , Manzella‐Lapeira J , Brzostowski J , Pierce SK (2020) Expression of inhibitory receptors by B cells in chronic human infectious diseases restricts responses to membrane‐associated antigens. Sci Adv 6: eaba6493 32754637 10.1126/sciadv.aba6493 PMC7380957

[emmm202217341-bib-0005] Anders H‐J , Saxena R , Zhao M‐H , Parodis I , Salmon JE , Mohan C (2020) Lupus nephritis. Nat Rev Dis Primers 6: 7 31974366 10.1038/s41572-019-0141-9

[emmm202217341-bib-0006] Badoual C (2022) Update from the 5th edition of the World Health Organization classification of head and neck tumors: oropharynx and nasopharynx. Head Neck Pathol 16: 19–30 35312986 10.1007/s12105-022-01449-2 PMC9019010

[emmm202217341-bib-0007] Batista FD , Harwood NE (2009) The who, how and where of antigen presentation to B cells. Nat Rev Immunol 9: 15–27 19079135 10.1038/nri2454

[emmm202217341-bib-0008] Beek MV , Nussenzweig MC , Chakraborty AK (2022) Two complementary features of humoral immune memory confer protection against the same or variant antigens. Proc Natl Acad Sci USA 119: e2205598119 36006981 10.1073/pnas.2205598119 PMC9477401

[emmm202217341-bib-0009] Bradley D , Smith AJ , Blaszczak A , Shantaram D , Bergin SM , Jalilvand A , Wright V , Wyne KL , Dewal RS , Baer LA *et al* (2022) Interferon gamma mediates the reduction of adipose tissue regulatory T cells in human obesity. Nat Commun 13: 5606 36153324 10.1038/s41467-022-33067-5 PMC9509397

[emmm202217341-bib-0010] Brown GJ , Cañete PF , Wang H , Medhavy A , Bones J , Roco JA , He Y , Qin Y , Cappello J , Ellyard JI *et al* (2022) TLR7 gain‐of‐function genetic variation causes human lupus. Nature 605: 349–356 35477763 10.1038/s41586-022-04642-z PMC9095492

[emmm202217341-bib-0011] Capolunghi F , Rosado MM , Sinibaldi M , Aranburu A , Carsetti R (2013) Why do we need IgM memory B cells? Immunol Lett 152: 114–120 23660557 10.1016/j.imlet.2013.04.007

[emmm202217341-bib-0012] Castleman MJ , Stumpf MM , Therrien NR , Smith MJ , Lesteberg KE , Palmer BE , Maloney JP , Janssen WJ , Mould KJ , Beckham JD *et al* (2022) Autoantibodies elicited with SARS‐CoV‐2 infection are linked to alterations in double negative B cells. Front Immunol 13: 988125 36131937 10.3389/fimmu.2022.988125 PMC9484582

[emmm202217341-bib-0013] Catlett IM , Bishop GA (1999) Cutting edge: a novel mechanism for rescue of B cells from CD95/Fas‐mediated apoptosis. J Immunol 163: 2378–2381 10452970

[emmm202217341-bib-0014] Centuori SM , Gomes CJ , Kim SS , Putnam CW , Larsen BT , Garland LL , Mount DW , Martinez JD (2018) Double‐negative (CD27^−^IgD^−^) B cells are expanded in NSCLC and inversely correlate with affinity‐matured B cell populations. J Transl Med 16: 30 29448960 10.1186/s12967-018-1404-z PMC5815250

[emmm202217341-bib-0015] Cervantes‐Díaz R , Sosa‐Hernández VA , Torres‐Ruíz J , Romero‐Ramírez S , Cañez‐Hernández M , Pérez‐Fragoso A , Páez‐Franco JC , Meza‐Sánchez DE , Pescador‐Rojas M , Sosa‐Hernández VA *et al* (2022) Severity of SARS‐CoV‐2 infection is linked to double‐negative (CD27^−^ IgD^−^) B cell subset numbers. Inflamm Res 71: 131–140 34850243 10.1007/s00011-021-01525-3 PMC8631699

[emmm202217341-bib-0016] Chen Z , Fillmore CM , Hammerman PS , Kim CF , Wong K‐K (2014) Non‐small‐cell lung cancers: a heterogeneous set of diseases. Nat Rev Cancer 14: 535–546 25056707 10.1038/nrc3775 PMC5712844

[emmm202217341-bib-0017] Chen J , Tan Y , Sun F , Hou L , Zhang C , Ge T , Yu H , Wu C , Zhu Y , Duan L *et al* (2020) Single‐cell transcriptome and antigen‐immunoglobin analysis reveals the diversity of B cells in non‐small cell lung cancer. Genome Biol 21: 152 32580738 10.1186/s13059-020-02064-6 PMC7315523

[emmm202217341-bib-0018] Cherukuri A , Cheng PC , Sohn HW , Pierce SK (2001) The CD19/CD21 complex functions to prolong B cell antigen receptor signaling from lipid rafts. Immunity 14: 169–179 11239449 10.1016/s1074-7613(01)00098-x

[emmm202217341-bib-0019] Colonna‐Romano G , Bulati M , Aquino A , Pellicanò M , Vitello S , Lio D , Candore G , Caruso C (2009) A double‐negative (IgD^−^CD27^−^) B cell population is increased in the peripheral blood of elderly people. Mech Ageing Dev 130: 681–690 19698733 10.1016/j.mad.2009.08.003

[emmm202217341-bib-0020] Cunningham AF , Gaspal F , Serre K , Mohr E , Henderson IR , Scott‐Tucker A , Kenny SM , Khan M , Toellner K‐M , Lane PJL *et al* (2007) Salmonella induces a switched antibody response without germinal centers that impedes the extracellular spread of infection. J Immunol 178: 6200–6207 17475847 10.4049/jimmunol.178.10.6200

[emmm202217341-bib-0021] De Silva NS , Klein U (2015) Dynamics of B cells in germinal centres. Nat Rev Immunol 15: 137–148 25656706 10.1038/nri3804 PMC4399774

[emmm202217341-bib-0022] Dengler HS , Baracho GV , Omori SA , Bruckner S , Arden KC , Castrillon DH , DePinho RA , Rickert RC (2008) Distinct functions for the transcription factor Foxo1 at various stages of B cell differentiation. Nat Immunol 9: 1388–1398 18978794 10.1038/ni.1667 PMC2679692

[emmm202217341-bib-0023] Dolff S , Abdulahad WH , Westra J , Doornbos‐van der Meer B , Limburg PC , Kallenberg CGM , Bijl M (2011) Increase in IL‐21 producing T‐cells in patients with systemic lupus erythematosus. Arthritis Res Ther 13: R157 21959034 10.1186/ar3474 PMC3308088

[emmm202217341-bib-0024] Eliopoulos AG , Stack M , Dawson CW , Kaye KM , Hodgkin L , Sihota S , Rowe M , Young LS (1997) Epstein‐Barr virus‐encoded LMP1 and CD40 mediate IL‐6 production in epithelial cells via an NF‐kappaB pathway involving TNF receptor‐associated factors. Oncogene 14: 2899–2916 9205097 10.1038/sj.onc.1201258

[emmm202217341-bib-0025] Elsner RA , Shlomchik MJ (2020) Germinal center and extrafollicular B cell responses in vaccination, immunity, and autoimmunity. Immunity 53: 1136–1150 33326765 10.1016/j.immuni.2020.11.006 PMC7748291

[emmm202217341-bib-0026] Fabrizi M , Marchetti V , Mavilio M , Marino A , Casagrande V , Cavalera M , Maria Moreno‐Navarrete J , Mezza T , Sorice GP , Fiorentino L *et al* (2014) IL‐21 is a major negative regulator of IRF4‐dependent lipolysis affecting Tregs in adipose tissue and systemic insulin sensitivity. Diabetes 63: 2086–2096 24430438 10.2337/db13-0939

[emmm202217341-bib-0027] Faustini F , Sippl N , Stålesen R , Chemin K , Dunn N , Fogdell‐Hahn A , Gunnarsson I , Malmström V (2022) Rituximab in systemic lupus erythematosus: transient effects on autoimmunity associated lymphocyte phenotypes and implications for immunogenicity. Front Immunol 13: 826152 35464461 10.3389/fimmu.2022.826152 PMC9027571

[emmm202217341-bib-0028] Fecteau JF , Cote G , Neron S (2006) A new memory CD27^−^IgG^+^ B cell population in peripheral blood expressing VH genes with low frequency of somatic mutation. J Immunol 177: 3728–3736 16951333 10.4049/jimmunol.177.6.3728

[emmm202217341-bib-0029] Franceschi C , Garagnani P , Parini P , Giuliani C , Santoro A (2018) Inflammaging: a new immune‐metabolic viewpoint for age‐related diseases. Nat Rev Endocrinol 14: 576–590 30046148 10.1038/s41574-018-0059-4

[emmm202217341-bib-0030] Franco A , Kraus Z , Li H , Seibert N , Dement‐Brown J , Tolnay M (2018) CD21 and FCRL5 form a receptor complex with robust B‐cell activating capacity. Int Immunol 30: 569–578 30107486 10.1093/intimm/dxy052

[emmm202217341-bib-0031] Franklin BS , Parroche P , Ataíde MA , Lauw F , Ropert C , de Oliveira RB , Pereira D , Tada MS , Nogueira P , da Silva LHP *et al* (2009) Malaria primes the innate immune response due to interferon‐γ induced enhancement of toll‐like receptor expression and function. Proc Natl Acad Sci USA 106: 5789–5794 19297619 10.1073/pnas.0809742106 PMC2657593

[emmm202217341-bib-0032] Frasca D , Landin AM , Lechner SC , Ryan JG , Schwartz R , Riley RL , Blomberg BB (2008) Aging down‐regulates the transcription factor E2A, activation‐induced cytidine deaminase, and Ig class switch in human B cells. J Immunol 180: 5283–5290 18390709 10.4049/jimmunol.180.8.5283

[emmm202217341-bib-0033] Frasca D , Diaz A , Romero M , Blomberg BB (2021) Phenotypic and functional characterization of double negative B cells in the blood of individuals with obesity. Front Immunol 12: 616650 33708209 10.3389/fimmu.2021.616650 PMC7940530

[emmm202217341-bib-0034] Golinski M‐L , Demeules M , Derambure C , Riou G , Maho‐Vaillant M , Boyer O , Joly P , Calbo S (2020) CD11c^+^ B cells are mainly memory cells, precursors of antibody secreting cells in healthy donors. Front Immunol 11: 32 32158442 10.3389/fimmu.2020.00032 PMC7051942

[emmm202217341-bib-0035] Gong L , Kwong DL‐W , Dai W , Wu P , Li S , Yan Q , Zhang Y , Zhang B , Fang X , Liu L *et al* (2021) Comprehensive single‐cell sequencing reveals the stromal dynamics and tumor‐specific characteristics in the microenvironment of nasopharyngeal carcinoma. Nat Commun 12: 1540 33750785 10.1038/s41467-021-21795-z PMC7943808

[emmm202217341-bib-0036] Gould HJ , Sutton BJ (2008) IgE in allergy and asthma today. Nat Rev Immunol 8: 205–217 18301424 10.1038/nri2273

[emmm202217341-bib-0037] Haga CL , Ehrhardt GR , Boohaker RJ , Davis RS , Cooper MD (2007) Fc receptor‐like 5 inhibits B cell activation via SHP‐1 tyrosine phosphatase recruitment. Proc Natl Acad Sci USA 104: 9770–9775 17522256 10.1073/pnas.0703354104 PMC1887609

[emmm202217341-bib-0038] Holla P , Dizon B , Ambegaonkar AA , Rogel N , Goldschmidt E , Boddapati AK , Sohn H , Sturdevant D , Austin JW , Kardava L *et al* (2021) Shared transcriptional profiles of atypical B cells suggest common drivers of expansion and function in malaria, HIV, and autoimmunity. Sci Adv 7: eabg8384 34039612 10.1126/sciadv.abg8384 PMC8153733

[emmm202217341-bib-0039] Hong S , Zhang Z , Liu H , Tian M , Zhu X , Zhang Z , Wang W , Zhou X , Zhang F , Ge Q *et al* (2018) B cells are the dominant antigen‐presenting cells that activate naive CD4^+^ T cells upon immunization with a virus‐derived nanoparticle antigen. Immunity 49: 695–708 30291027 10.1016/j.immuni.2018.08.012

[emmm202217341-bib-0040] Hopp CS , Skinner J , Anzick SL , Tipton CM , Peterson ME , Li S , Doumbo S , Kayentao K , Ongoiba A , Martens C *et al* (2022) Atypical B cells up‐regulate costimulatory molecules during malaria and secrete antibodies with T follicular helper cell support. Sci Immunol 7: eabn1250 35559666 10.1126/sciimmunol.abn1250 PMC11132112

[emmm202217341-bib-0041] Hritzo Ahye MK , Golding A (2018) Cytoplasmic FOXO1 identifies a novel disease‐activity associated B cell phenotype in SLE. Lupus Sci Med 5: e000296 30397498 10.1136/lupus-2018-000296 PMC6203050

[emmm202217341-bib-0042] Hu B , Guo H , Zhou P , Shi Z‐L (2021) Characteristics of SARS‐CoV‐2 and COVID‐19. Nat Rev Microbiol 19: 141–154 33024307 10.1038/s41579-020-00459-7 PMC7537588

[emmm202217341-bib-0043] Huang C , Wang Y , Li X , Ren L , Zhao J , Hu Y , Zhang L , Fan G , Xu J , Gu X *et al* (2020) Clinical features of patients infected with 2019 novel coronavirus in Wuhan, China. Lancet 395: 497–506 31986264 10.1016/S0140-6736(20)30183-5 PMC7159299

[emmm202217341-bib-0044] Jacobi AM , Reiter K , Mackay M , Aranow C , Hiepe F , Radbruch A , Hansen A , Burmester GR , Diamond B , Lipsky PE *et al* (2008) Activated memory B cell subsets correlate with disease activity in systemic lupus erythematosus: delineation by expression of CD27, IgD, and CD95. Arthritis Rheum 58: 1762–1773 18512812 10.1002/art.23498

[emmm202217341-bib-0045] Jenks SA , Cashman KS , Zumaquero E , Marigorta UM , Patel AV , Wang X , Tomar D , Woodruff MC , Simon Z , Bugrovsky R *et al* (2018) Distinct effector B cells induced by unregulated toll‐like receptor 7 contribute to pathogenic responses in systemic lupus erythematosus. Immunity 49: 725–739 30314758 10.1016/j.immuni.2018.08.015 PMC6217820

[emmm202217341-bib-0046] Jenks SA , Cashman KS , Woodruff MC , Lee FEH , Sanz I (2019) Extrafollicular responses in humans and SLE. Immunol Rev 288: 136–148 30874345 10.1111/imr.12741 PMC6422038

[emmm202217341-bib-0047] Jenks SA , Wei C , Bugrovsky R , Hill A , Wang X , Rossi FM , Cashman K , Woodruff MC , Aspey LD , Lim SS *et al* (2021) B cell subset composition segments clinically and serologically distinct groups in chronic cutaneous lupus erythematosus. Ann Rheum Dis 80: 1190–1200 34083207 10.1136/annrheumdis-2021-220349 PMC8906255

[emmm202217341-bib-0048] Kaul A , Gordon C , Crow MK , Touma Z , Urowitz MB , Ruiz‐Irastorza G , Hughes G , van Vollenhoven R (2016) Systemic lupus erythematosus. Nat Rev Dis Primers 2: 16039 27306639 10.1038/nrdp.2016.39

[emmm202217341-bib-0049] Kazanietz MG , Durando M , Cooke M (2019) CXCL13 and its receptor CXCR5 in cancer: inflammation, immune response, and beyond. Front Endocrinol (Lausanne) 10: 471 31354634 10.3389/fendo.2019.00471 PMC6639976

[emmm202217341-bib-0050] Kim CC , Baccarella AM , Bayat A , Pepper M , Fontana MF (2019) FCRL5^+^ memory B cells exhibit robust recall responses. Cell Rep 27: 1446–1460 31042472 10.1016/j.celrep.2019.04.019 PMC6530801

[emmm202217341-bib-0051] Klein U , Goossens T , Fischer M , Kanzler H , Braeuninger A , Rajewsky K , Küppers R (1998) Somatic hypermutation in normal and transformed human B cells. Immunol Rev 162: 261–280 9602370 10.1111/j.1600-065x.1998.tb01447.x

[emmm202217341-bib-0052] KleinJan A , Vinke JG , Severijnen LW , Fokkens WJ (2000) Local production and detection of (specific) IgE in nasal B‐cells and plasma cells of allergic rhinitis patients. Eur Respir J 15: 491–497 10759442 10.1034/j.1399-3003.2000.15.11.x

[emmm202217341-bib-0053] Kometani K , Nakagawa R , Shinnakasu R , Kaji T , Rybouchkin A , Moriyama S , Furukawa K , Koseki H , Takemori T , Kurosaki T (2013) Repression of the transcription factor Bach2 contributes to predisposition of IgG1 memory B cells toward plasma cell differentiation. Immunity 39: 136–147 23850379 10.1016/j.immuni.2013.06.011

[emmm202217341-bib-0054] Koon H‐K , Lo K‐W , Leung K‐N , Lung ML , Chang CC‐K , Wong RN‐S , Leung W‐N , Mak N‐K (2010) Photodynamic therapy‐mediated modulation of inflammatory cytokine production by Epstein‐Barr virus‐infected nasopharyngeal carcinoma cells. Cell Mol Immunol 7: 323–326 20228836 10.1038/cmi.2010.4 PMC4003233

[emmm202217341-bib-0055] Kuri‐Cervantes L , Pampena MB , Meng W , Rosenfeld AM , Ittner CAG , Weisman AR , Agyekum RS , Mathew D , Baxter AE , Vella LA *et al* (2020) Comprehensive mapping of immune perturbations associated with severe COVID‐19. Sci Immunol 5: eabd7114 32669287 10.1126/sciimmunol.abd7114 PMC7402634

[emmm202217341-bib-0056] Lee SK , Rigby RJ , Zotos D , Tsai LM , Kawamoto S , Marshall JL , Ramiscal RR , Chan TD , Gatto D , Brink R *et al* (2011) B cell priming for extrafollicular antibody responses requires Bcl‐6 expression by T cells. J Exp Med 208: 1377–1388 21708925 10.1084/jem.20102065 PMC3135363

[emmm202217341-bib-0057] Li Y , Li Z , Hu F (2021) Double‐negative (DN) B cells: an under‐recognized effector memory B cell subset in autoimmunity. Clin Exp Immunol 205: 119–127 33969476 10.1111/cei.13615 PMC8274172

[emmm202217341-bib-0058] Liu C , Richard K , Wiggins M , Zhu X , Conrad DH , Song W (2016) CD23 can negatively regulate B‐cell receptor signaling. Sci Rep 6: 25629 27181049 10.1038/srep25629 PMC4867583

[emmm202217341-bib-0059] Lortan JE , Vellodi A , Jurges ES , Hugh‐Jones K (1992) Class‐ and subclass‐specific pneumococcal antibody levels and response to immunization after bone marrow transplantation. Clin Exp Immunol 88: 512–519 1606736 10.1111/j.1365-2249.1992.tb06480.x PMC1554526

[emmm202217341-bib-0060] Lund JM , Alexopoulou L , Sato A , Karow M , Adams NC , Gale NW , Iwasaki A , Flavell RA (2004) Recognition of single‐stranded RNA viruses by toll‐like receptor 7. Proc Natl Acad Sci USA 101: 5598–5603 15034168 10.1073/pnas.0400937101 PMC397437

[emmm202217341-bib-0061] Mäkelä O , Rouslahti E , Seppälä IJT (1970) Affinity of IgM and IgG antibodies. Immunochemistry 7: 917–932 4992788 10.1016/0019-2791(70)90053-4

[emmm202217341-bib-0062] Manoussakis MN , Tzioufas AG , Silis MP , Pange PJE , Goudevenos J , Moutsopoulos HM (1987) High prevalence of anti‐cardiolipin and other autoantibodies in a healthy elderly population. Clin Exp Immunol 69: 557–565 3499270 PMC1542366

[emmm202217341-bib-0063] McHeyzer‐Williams LJ , Milpied PJ , Okitsu SL , McHeyzer‐Williams MG (2015) Class‐switched memory B cells remodel BCRs within secondary germinal centers. Nat Immunol 16: 296–305 25642821 10.1038/ni.3095 PMC4333102

[emmm202217341-bib-0064] Mensah FFK , Armstrong CW , Reddy V , Bansal AS , Berkovitz S , Leandro MJ , Cambridge G (2018) CD24 expression and B cell maturation shows a novel link with energy metabolism: potential implications for patients with myalgic encephalomyelitis/chronic fatigue syndrome. Front Immunol 9: 2421 30405620 10.3389/fimmu.2018.02421 PMC6204382

[emmm202217341-bib-0065] Moran I , Nguyen A , Khoo WH , Butt D , Bourne K , Young C , Hermes JR , Biro M , Gracie G , Ma CS *et al* (2018) Memory B cells are reactivated in subcapsular proliferative foci of lymph nodes. Nat Commun 9: 3372–3314 30135429 10.1038/s41467-018-05772-7 PMC6105623

[emmm202217341-bib-0066] Muehlinghaus G , Cigliano L , Huehn S , Peddinghaus A , Leyendeckers H , Hauser AE , Hiepe F , Radbruch A , Arce S , Manz RA (2005) Regulation of CXCR3 and CXCR4 expression during terminal differentiation of memory B cells into plasma cells. Blood 105: 3965–3971 15687242 10.1182/blood-2004-08-2992

[emmm202217341-bib-0067] Muramatsu M , Kinoshita K , Fagarasan S , Yamada S , Shinkai Y , Honjo T (2000) Class switch recombination and hypermutation require activation‐induced cytidine deaminase (AID), a potential RNA editing enzyme. Cell 102: 553–563 11007474 10.1016/s0092-8674(00)00078-7

[emmm202217341-bib-0068] Oke V , Gunnarsson I , Dorschner J , Eketjäll S , Zickert A , Niewold TB , Svenungsson E (2019) High levels of circulating interferons type I, type II and type III associate with distinct clinical features of active systemic lupus erythematosus. Arthritis Res Ther 21: 107 31036046 10.1186/s13075-019-1878-y PMC6489203

[emmm202217341-bib-0069] Peng S , Szabo S , Glimcher L (2002) T‐bet regulates IgG class switching and pathogenic autoantibody production. Proc Natl Acad Sci USA 99: 5545–5550 11960012 10.1073/pnas.082114899 PMC122806

[emmm202217341-bib-0070] Phillips MA , Burrows JN , Manyando C , van Huijsduijnen RH , Van Voorhis WC , Wells TNC (2017) Malaria. Nat Rev Dis Primers 3: 17050 28770814 10.1038/nrdp.2017.50

[emmm202217341-bib-0071] Pioli PD (2019) Plasma cells, the next generation: beyond antibody secretion. Front Immunol 10: 2768 31824518 10.3389/fimmu.2019.02768 PMC6883717

[emmm202217341-bib-0072] Portugal S , Tipton CM , Sohn H , Kone Y , Wang J , Li S , Skinner J , Virtaneva K , Sturdevant DE , Porcella SF *et al* (2015) Malaria‐associated atypical memory B cells exhibit markedly reduced B cell receptor signaling and effector function. Elife 4: e07218 25955968 10.7554/eLife.07218 PMC4444601

[emmm202217341-bib-0073] Reilly SM , Saltiel AR (2017) Adapting to obesity with adipose tissue inflammation. Nat Rev Endocrinol 13: 633–643 28799554 10.1038/nrendo.2017.90

[emmm202217341-bib-0074] Revelo Xavier S , Ghazarian M , Chng Melissa Hui Y , Luck H , Kim Justin H , Zeng K , Shi Sally Y , Tsai S , Lei H , Kenkel J *et al* (2016) Nucleic acid‐targeting pathways promote inflammation in obesity‐related insulin resistance. Cell Rep 16: 717–730 27373163 10.1016/j.celrep.2016.06.024 PMC6354586

[emmm202217341-bib-0075] Rojas M , Rodríguez Y , Acosta‐Ampudia Y , Monsalve DM , Zhu C , Li Q‐Z , Ramírez‐Santana C , Anaya J‐M (2022) Autoimmunity is a hallmark of post‐COVID syndrome. J Transl Med 20: 129 35296346 10.1186/s12967-022-03328-4 PMC8924736

[emmm202217341-bib-0076] Rolink AG , Schaniel C , Andersson J , Melchers F (2001) Selection events operating at various stages in B cell development. Curr Opin Immunol 13: 202–207 11228414 10.1016/s0952-7915(00)00205-3

[emmm202217341-bib-0077] Rubtsov AV , Rubtsova K , Fischer A , Meehan RT , Gillis JZ , Kappler JW , Marrack P (2011) Toll‐like receptor 7 (TLR7)‐driven accumulation of a novel CD11c^+^ B‐cell population is important for the development of autoimmunity. Blood 118: 1305–1315 21543762 10.1182/blood-2011-01-331462 PMC3152497

[emmm202217341-bib-0078] Rubtsov AV , Rubtsova K , Kappler JW , Jacobelli J , Friedman RS , Marrack P (2015) CD11c‐expressing B cells are located at the T cell/B cell border in spleen and are potent APCs. J Immunol 195: 71–79 26034175 10.4049/jimmunol.1500055 PMC4475418

[emmm202217341-bib-0079] Russo L , Lumeng CN (2018) Properties and functions of adipose tissue macrophages in obesity. Immunology 155: 407–417 30229891 10.1111/imm.13002 PMC6230999

[emmm202217341-bib-0080] Sanz I , Wei C , Jenks SA , Cashman KS , Tipton C , Woodruff MC , Hom J , Lee FE (2019) Challenges and opportunities for consistent classification of human B cell and plasma cell populations. Front Immunol 10: 2458 31681331 10.3389/fimmu.2019.02458 PMC6813733

[emmm202217341-bib-0081] Sato S (2021) Plasmodium‐a brief introduction to the parasites causing human malaria and their basic biology. J Physiol Anthropol 40: 1 33413683 10.1186/s40101-020-00251-9 PMC7792015

[emmm202217341-bib-0082] Schultheiss C , Paschold L , Willscher E , Simnica D , Wostemeier A , Muscate F , Wass M , Eisenmann S , Dutzmann J , Keysser G *et al* (2021) Maturation trajectories and transcriptional landscape of plasmablasts and autoreactive B cells in COVID‐19. iScience 24: 103325 34723157 10.1016/j.isci.2021.103325 PMC8536484

[emmm202217341-bib-0083] Seifert M , Przekopowitz M , Taudien S , Lollies A , Ronge V , Drees B , Lindemann M , Hillen U , Engler H , Singer BB *et al* (2015) Functional capacities of human IgM memory B cells in early inflammatory responses and secondary germinal center reactions. Proc Natl Acad Sci USA 112: E546–E555 25624468 10.1073/pnas.1416276112 PMC4330750

[emmm202217341-bib-0084] Song W , Antao OQ , Condiff E , Sanchez GM , Chernova I , Zembrzuski K , Steach H , Rubtsova K , Angeletti D , Lemenze A *et al* (2022) Development of Tbet‐ and CD11c‐expressing B cells in a viral infection requires T follicular helper cells outside of germinal centers. Immunity 55: 290–307 35090581 10.1016/j.immuni.2022.01.002 PMC8965751

[emmm202217341-bib-0085] Sosa‐Hernández VA , Torres‐Ruíz J , Cervantes‐Díaz R , Romero‐Ramírez S , Páez‐Franco JC , Meza‐Sánchez DE , Juárez‐Vega G , Pérez‐Fragoso A , Ortiz‐Navarrete V , Ponce‐de‐León A *et al* (2020) B cell subsets as severity‐associated signatures in COVID‐19 patients. Front Immunol 11: 611004 33343585 10.3389/fimmu.2020.611004 PMC7744304

[emmm202217341-bib-0086] Souyris M , Cenac C , Azar P , Daviaud D , Canivet A , Grunenwald S , Pienkowski C , Chaumeil J , Mejía JE , Guéry J‐C (2018) TLR7 escapes X chromosome inactivation in immune cells. Sci Immunol 3: eaap8855 29374079 10.1126/sciimmunol.aap8855

[emmm202217341-bib-0087] Stewart A , Ng JC , Wallis G , Tsioligka V , Fraternali F , Dunn‐Walters DK (2021) Single‐cell transcriptomic analyses define distinct peripheral B cell subsets and discrete development pathways. Front Immunol 12: 602539 33815362 10.3389/fimmu.2021.602539 PMC8012727

[emmm202217341-bib-0088] Sutton HJ , Aye R , Idris AH , Vistein R , Nduati E , Kai O , Mwacharo J , Li X , Gao X , Andrews TD *et al* (2021) Atypical B cells are part of an alternative lineage of B cells that participates in responses to vaccination and infection in humans. Cell Rep 34: 108684 33567273 10.1016/j.celrep.2020.108684 PMC7873835

[emmm202217341-bib-0089] Toellner KM , Luther SA , Sze DM , Choy RK , Taylor DR , MacLennan IC , Acha‐Orbea H (1998) T helper 1 (Th1) and Th2 characteristics start to develop during T cell priming and are associated with an immediate ability to induce immunoglobulin class switching. J Exp Med 187: 1193–1204 9547331 10.1084/jem.187.8.1193 PMC2212236

[emmm202217341-bib-0090] Torigoe M , Iwata S , Nakayamada S , Sakata K , Zhang M , Hajime M , Miyazaki Y , Narisawa M , Ishii K , Shibata H *et al* (2017) Metabolic reprogramming commits differentiation of human CD27^+^IgD^+^ B cells to plasmablasts or CD27^−^IgD^−^ cells. J Immunol 199: 425–434 28626065 10.4049/jimmunol.1601908

[emmm202217341-bib-0091] Tran TM , Li S , Doumbo S , Doumtabe D , Huang C‐Y , Dia S , Bathily A , Sangala J , Kone Y , Traore A *et al* (2013) An intensive longitudinal cohort study of Malian children and adults reveals no evidence of acquired immunity to *Plasmodium falciparum* infection. Clin Infect Dis 57: 40–47 23487390 10.1093/cid/cit174 PMC3669526

[emmm202217341-bib-0092] Tsao SW , Tsang CM , Lo KW (2017) Epstein‐Barr virus infection and nasopharyngeal carcinoma. Philos Trans R Soc Lond B Biol Sci 372: 20160270 28893937 10.1098/rstb.2016.0270 PMC5597737

[emmm202217341-bib-0093] V'Kovski P , Kratzel A , Steiner S , Stalder H , Thiel V (2021) Coronavirus biology and replication: implications for SARS‐CoV‐2. Nat Rev Microbiol 19: 155–170 33116300 10.1038/s41579-020-00468-6 PMC7592455

[emmm202217341-bib-0094] Wang Y , Fu Z , Li X , Liang Y , Pei S , Hao S , Zhu Q , Yu T , Pei Y , Yuan J *et al* (2021) Cytoplasmic DNA sensing by KU complex in aged CD4^+^ T cell potentiates T cell activation and aging‐related autoimmune inflammation. Immunity 54: 632–647 33667382 10.1016/j.immuni.2021.02.003

[emmm202217341-bib-0095] Wei C , Anolik J , Cappione A , Zheng B , Pugh‐Bernard A , Brooks J , Lee E‐H , Milner ECB , Sanz I (2007) A new population of cells lacking expression of CD27 represents a notable component of the B cell memory compartment in systemic lupus erythematosus. J Immunol 178: 6624–6633 17475894 10.4049/jimmunol.178.10.6624

[emmm202217341-bib-0096] William J , Euler C , Christensen S , Shlomchik MJ (2002) Evolution of autoantibody responses via somatic hypermutation outside of germinal centers. Science 297: 2066–2070 12242446 10.1126/science.1073924

[emmm202217341-bib-0097] Winer DA , Winer S , Shen L , Wadia PP , Yantha J , Paltser G , Tsui H , Wu P , Davidson MG , Alonso MN *et al* (2011) B cells promote insulin resistance through modulation of T cells and production of pathogenic IgG antibodies. Nat Med 17: 610–617 21499269 10.1038/nm.2353 PMC3270885

[emmm202217341-bib-0098] Winer DA , Winer S , Chng MH , Shen L , Engleman EG (2014) B lymphocytes in obesity‐related adipose tissue inflammation and insulin resistance. Cell Mol Life Sci 71: 1033–1043 24127133 10.1007/s00018-013-1486-y PMC3954849

[emmm202217341-bib-0099] Wong KCW , Hui EP , Lo K‐W , Lam WKJ , Johnson D , Li L , Tao Q , Chan KCA , To K‐F , King AD *et al* (2021) Nasopharyngeal carcinoma: an evolving paradigm. Nat Rev Clin Oncol 18: 679–695 34194007 10.1038/s41571-021-00524-x

[emmm202217341-bib-0100] Woodruff MC , Ramonell RP , Nguyen DC , Cashman KS , Saini AS , Haddad NS , Ley AM , Kyu S , Howell JC , Ozturk T *et al* (2020) Extrafollicular B cell responses correlate with neutralizing antibodies and morbidity in COVID‐19. Nat Immunol 21: 1506–1516 33028979 10.1038/s41590-020-00814-z PMC7739702

[emmm202217341-bib-0101] Woodruff MC , Ramonell RP , Haddad NS , Anam FA , Rudolph ME , Walker TA , Truong AD , Dixit AN , Han JE , Cabrera‐Mora M *et al* (2022) Dysregulated naïve B cells and *de novo* autoreactivity in severe COVID‐19. Nature 611: 139–147 36044993 10.1038/s41586-022-05273-0 PMC9630115

[emmm202217341-bib-0102] Wu YC , Kipling D , Dunn‐Walters DK (2011) The relationship between CD27 negative and positive B cell populations in human peripheral blood. Front Immunol 2: 81 22566870 10.3389/fimmu.2011.00081 PMC3341955

[emmm202217341-bib-0103] Wu J , Wu H , An J , Ballantyne CM , Cyster JG (2018) Critical role of integrin CD11c in splenic dendritic cell capture of missing‐self CD47 cells to induce adaptive immunity. Proc Natl Acad Sci USA 115: 6786–6791 29891680 10.1073/pnas.1805542115 PMC6042080

[emmm202217341-bib-0104] Yang L , Xie X , Tu Z , Fu J , Xu D , Zhou Y (2021) The signal pathways and treatment of cytokine storm in COVID‐19. Signal Transduct Target Ther 6: 255 34234112 10.1038/s41392-021-00679-0 PMC8261820

[emmm202217341-bib-0105] You X , Zhang R , Shao M , He J , Chen J , Liu J , Zhang X , Liu X , Jia R , Sun X *et al* (2020) Double negative B cell is associated with renal impairment in systemic lupus erythematosus and acts as a marker for nephritis remission. Front Med (Lausanne) 7: 85 32318574 10.3389/fmed.2020.00085 PMC7155774

[emmm202217341-bib-0106] Yu B , Qi Y , Li R , Shi Q , Satpathy AT , Chang HY (2021) B cell‐specific XIST complex enforces X‐inactivation and restrains atypical B cells. Cell 184: 1790–1803 33735607 10.1016/j.cell.2021.02.015 PMC9196326

[emmm202217341-bib-0107] Zatterale F , Longo M , Naderi J , Raciti GA , Desiderio A , Miele C , Beguinot F (2019) Chronic adipose tissue inflammation linking obesity to insulin resistance and type 2 diabetes. Front Physiol 10: 1607 32063863 10.3389/fphys.2019.01607 PMC7000657

